# Improved Differential Evolution Algorithm for Sensitivity Enhancement of Surface Plasmon Resonance Biosensor Based on Two-Dimensional Material for Detection of Waterborne Bacteria

**DOI:** 10.3390/bios13060600

**Published:** 2023-05-31

**Authors:** Lei Han, Wentao Xu, Tao Liu, Yong Zhang, Yanhua Ma, Min Jin, Chaoyu Xu

**Affiliations:** 1 College of Mechanical and Electrical Engineering, Inner Mongolia Agricultural University, Hohhot 010018, China; xwt13131454520@163.com (W.X.); liutao@imau.edu.cn (T.L.); zynmg@imau.edu.cn (Y.Z.); mayanhua@imau.edu.cn (Y.M.); 15849345010@imau.edu.cn (M.J.); 2 School of Mechanical Engineering and Electronic Information, China University of Geosciences (Wuhan), Wuhan 430074, China; xuchaoyu1990@163.com

**Keywords:** surface plasmon resonance, MXene, graphene, waterborne bacteria, sensitivity, improved differential evolution

## Abstract

Due to the large number of waterborne bacteria presenting in drinking water, their rapid and accurate identification has become a global priority. The surface plasmon resonance (SPR) biosensor with prism (BK7)-silver(Ag)-MXene(Ti_3_T_2_Cx)-graphene- affinity-sensing medium is examined in this paper, in which the sensing medium includes pure water, vibrio cholera (*V. cholera*), and escherichia coli (*E. coli*). For the Ag-affinity-sensing medium, the maximum sensitivity is obtained by *E. coli*, followed by *V. cholera*, and the minimum is pure water. Based on the fixed-parameter scanning (FPS) method, the highest sensitivity is 246.2 °/RIU by the MXene and graphene with monolayer, and with *E. coli* sensing medium. Therefore, the algorithm of improved differential evolution (IDE) is obtained. By the IDE algorithm, after three iterations, the maximum fitness value (sensitivity) of the SPR biosensor achieves 246.6 °/RIU by using the structure of Ag (61 nm)-MXene (monolayer)-graphene (monolayer)-affinity (4 nm)-*E. coli*. Compared with the FPS and differential evolution (DE) algorithm, the highest sensitivity is more accurate and efficient, and with fewer iterations. The performance optimization of multilayer SPR biosensors provides an efficient platform.

## 1. Introduction

The surface plasmon resonance (SPR) biosensor is also an optical refractometer, which can be used to measure the refractive index change of the dielectric material on the SPR sensing surface. The working principle of this SRP biosensor is based on a unique and simple optical phenomenon, that is, in the precious metal conduction band which is the sensitive part, the free electrons undergo a coherent oscillation of the set. Firstly, this oscillation occurs at the metal/dielectric interface due to the interaction caused by the excitation of incident light (electromagnetic wave) [1]. These oscillations of charge density established by resonance can be called surface plasmon polaritons (SPPs). These SPPs will then form an exponentially decaying electric field, penetrating the surrounding medium with a depth of about a few micrometers [2]. When the refractive index of the sensitive medium changes, the characteristics of the incident light used to excite the SPR (such as angle, wavelength, phase, etc.) will also change accordingly, so it can perceive the existence of exotic species that cause the refractive index change of the medium to achieve the purpose of detection. Due to the sensitivity and dynamic response of the refractive index of the medium attached to the metal surface, it has applications in real-time detection of varieties, biomedical diagnosis [3,4,5], environmental monitoring [6,7], food industry [8,9] and other fields.

SPR detection technology has outstanding advantages: (1) label-free [10]: SPR technology can be combined by the specific recognition of ligands and receptors, so as to realize the recognition of target substances, without labeling specific substances, thus maximizing the in-situ nature of the sample; (2) miniaturized multi-point detection [11]: the SPR biosensor can realize miniaturization, can facilitate multi-point monitoring, is suitable for manufacturing portable biosensors, and can adapt to clinical and multi-point environmental monitoring. However, the traditional metal SPR biosensor has the problems of low detection sensitivity, poor accuracy, sensitivity to sample composition and temperature and other interference factors, and difficulty distinguishing non-specific adsorption [12]. The development trend of SPR biosensors is gradually improving the sensing material, improving the detection sensitivity [13], developing array and multi-channel SPR detection instruments [14], and developing towards miniaturization and portability [15]. 

In recent years, two-dimensional (2D) materials have included transition metal dichalcogenides (TMDCs) [16], MXene [17], graphene [18], black phosphorus (BP) [19], blue phosphorus (BlueP) [20], among others. Wu et al. [18] proved by the numerical method that the addition of graphene on the Au surface can be improved, and the sensitivity enhancement was calculated as (1 + 0.025 *L*) × *γ*, where *γ* represents the increase of adsorption of biological molecules on graphene and *L* represents the number of graphene layers. Chen et al. [21] studied the wavelength modulation of Au-MoS_2_ SPR biosensor. Their results show that MoS_2_ is three layers, and a maximum sensitivity of 2793.5 nm/RIU was obtained, which was 30.67% higher than that without any modification. The results show that the increase of TMDCs coating can improve the strength of the electric field penetrating the analytic solution and shorten the propagation length, resulting in the non-monotonic change of sensitivity with the deposition period. Yi et al. [22] proposed a new SPR sensor, which is composed of an Au-TMDCs-Au-MXene hybrid structure. Srivastava et al. [23] designed a novel SPR sensor including an Au-MXene-WS_2_-BP hybrid structure. The highest sensitivity of 190.22 °/RIU is obtained using a monolayer of each nanomaterial. Wu et al. [24] proposed the SPR biosensor with Au-MXene (Ti_3_C_2_Tx) structure. The sensitivity results of the MXene monolayer biosensor can reach 224.5°/RIU at the incident wavelength of 532 nm. Zhang et al. [25] proposed the Au-Ag-Si- MXene hybrid of SPR sensor structure. When the MXene with monolayer is obtained, the sensitivity is 274°/RIU. According to the above analysis, graphene and MXene can significantly improve the sensitivity of the SPR biosensor. All the above calculations were performed using fixed variable parameter scanning. 

FPS is time-consuming and it is difficult to find the optimal configuration. Therefore, an intelligent analysis and optimization platform for multi-layer SPR biosensors has been developed. The genetic algorithm (GA) is also applied to the optimization and improvement of SPR biosensor performance. Lin et al. [26,27] introduced the constraints of sensitivity and resonance angle reflectivity into the fitness function of GA and designed a sensing structure based on Au-Ag- TiO_2_-graphene. The thickness of the metal and the 2D material were optimized by GA to the incident light at a specific wavelength and optimal sensitivity [28]. Xia et al. [29] proposed GA to optimize the thickness of SPR multilayers, which is mainly used to optimize the thickness of 2D materials, effectively finding the best film structure and improving the sensitivity. However, when using the GA it is easy to fall into non-standardized coding, inaccuracies, and premature phenomena, and it is easy to fall into the local optimal solution and not easy to jump out, while the number of iterations increases [27]. The basic concept of the particle swarm optimization (PSO) algorithm is to provide a particle swarm moving in the problem space and to evaluate its optimal position through a fitness function [30]. Sun et al. [31] proposed an optimization of SPR biosensors based on the PSO algorithm and studied the sensing performance optimization of structural multi-parameter SPR sensors under four modulation modes (phase, strength, wavelength, and angle). The experimental structure and the corresponding optimization structure are compared, and the PSO algorithm has high efficiency. Amoosoltani et al. [32] studied an SPR-based optical gas sensor based on a PSO algorithm, and optimized the performance of the sensor from different angles such as metal layer thickness, prism, incident light angle, and wavelength. We propose an improved PSO algorithm with a high global optimal solution convergence rate [33]. Based on the improved PSO algorithm, the TMDCs and graphene composite SPR gas sensor were proposed and optimized. With the standard PSO algorithm, the number of iterations is reduced and the efficiency is increased. The PSO algorithm does not deal well with discrete optimization problems and is easy to fall into local optimum [34]. Therefore, a simple and efficient random heuristic search algorithm is proposed for the differential evolution (DE) algorithm. DE algorithm adopts real number coding, which has the advantages of simple principle, easy programming, few parameters, fast convergence, strong robustness, strong global optimization ability, and high optimization efficiency [35]. The DE algorithm is also an intelligent optimization algorithm, which is similar to the previous heuristic algorithms, such as GA, PSO, and so on. DE algorithm is an optimization algorithm used in a paper to solve the box coverage problem [36]. However, since the key step of differential evolution is to modify the value of each individual based on the difference vector information of the group, with the increase of evolutionary generations, the difference information between individuals is gradually narrowing, so that the convergence rate becomes slow in the late stage, sometimes falling into local optimum. Han et al. proposed a new DE algorithm based on the characteristics of SPR sensor multi-layer materials, which increased the Goos–Hänchen shift by 192 times [37]. 

Therefore, in this paper, the SPR biosensor with Ag-MXene-graphene-affinity-sensing medium is proposed for the detection of waterborne bacteria. The performance of the SPR biosensor is optimized by the improved differential evolution (IDE) algorithm. The multi-layer SPR biosensor greatly improves the overall performance efficiency and reduces the time.

## 2. Design Configuration and Theoretical Method

**Figure 1:**
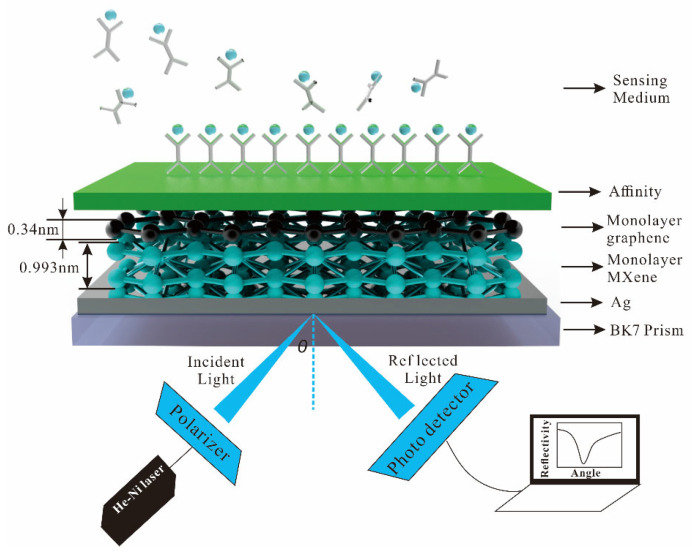
Schematic diagram of the SPR biosensor for sensitivity.

The heterostructure of the 2D material used in this paper improves the sensitivity, and an N-layer SPR biosensor with Kretschmann configuration is proposed. The He-Ne laser of wavelength 632.8 nm is fixed as p-polarized light. For the SPR biosensor, all layers are stacked perpendicular to a prism, refractive index (*n*_k_) and dielectric constant (*ε*_k_ = *n*_k_^2^), and the thickness (*d*_k_) of each layer. The angle between the incident light and the normal of the incident surface is called the incident angle. In optical refraction, the sine of the incident angle is equal to the sine of the refraction angle multiplied by the refractive index. When the light enters the optically sparse medium from the optically dense medium, the total reflection phenomenon can occur when the incident angle is greater than the critical angle. Phase refers to the offset or delay of the waveform relative to a reference point or reference signal. The simple sine wave usually refers to the offset between the waveform and the starting point of time. The schematic diagram of the SPR biosensor is illustrated in Figure 1. The N-layer of the SPR biosensor consists of BK7 glass as coupling prism (*n*_1_ = 1.515) [38], Ag (*n*_2_ = 0.135 + 3.985*i*) [39], 2D materials (MXene, graphene) as shown in Table 1 [25,40], affinity (nicotine, *n*_5_ = 1.5265) [41], and sensing medium (pure water, vibrio cholera (*V. cholera*), and escherichia coli (*E. coli*)) as Table 2 [42,43,44].

The transfer matrix method (TMM) and the Fresnel equation analyze the reflectance of the *N*-layer model of incident TM-polarized light. The detailed calculation process is shown in references [38,40].

**Figure 2:**
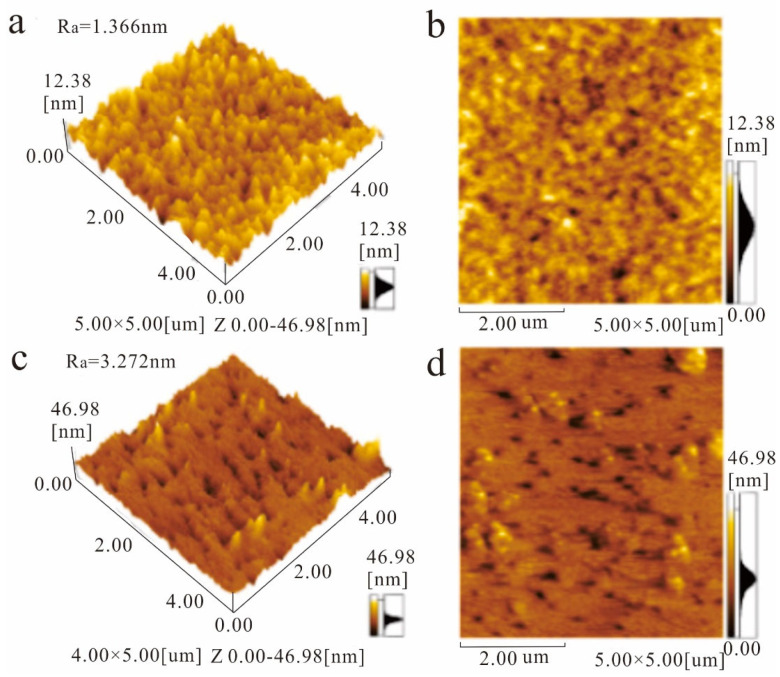
AFM images of 3D morphology (**a**) and 2D morphology (**b**) of monolayer graphene film, 3D morphology (**c**), and 2D morphology (**d**) of MXene/graphene composite film.

We use atomic force microscopy (AFM). The surface morphology of graphene and MXene/graphene composite films was further characterized by AFM. As shown in Figure 2a,b, the average roughness of the monolayer graphene film is 1.366 nm, and the film is smooth and continuous. Figure 2c,d show the AFM image of the MXene/graphene composite film. The average roughness of the composite film is 3.272 nm, and the film is flat and continuous.

The preparation method of graphene on the MXene monolayer is as follows. (1) The raw material Ti and AlC_2_ are chemically etched with lithium fluoride (LiF) and hydrochloric acid (HC_1_) as etchants, and the organ-strong MXene phase was obtained by stirring etching. The monolayer MXene nano-uniform aqueous solution was prepared by ultrasonic stripping and centrifugation. (2) Monolayer MXene nanosheets were prepared by ultrasonic stripping and centrifugal separation. The uniform aqueous solution of monolayer MXene nanosheets was added to the graphene oxide (GO) aqueous solution for ultrasonic treatment to form a uniform dispersion of monolayer MXene nanosheets and planned graphene. The monolayer MXene and GO nanosheets in the uniform dispersion were self-assembled to obtain MXene functionalized graphene oxide composites.

## 3. Optimization of the SPR Biosensor Structure Parameters

**Figure 3:**
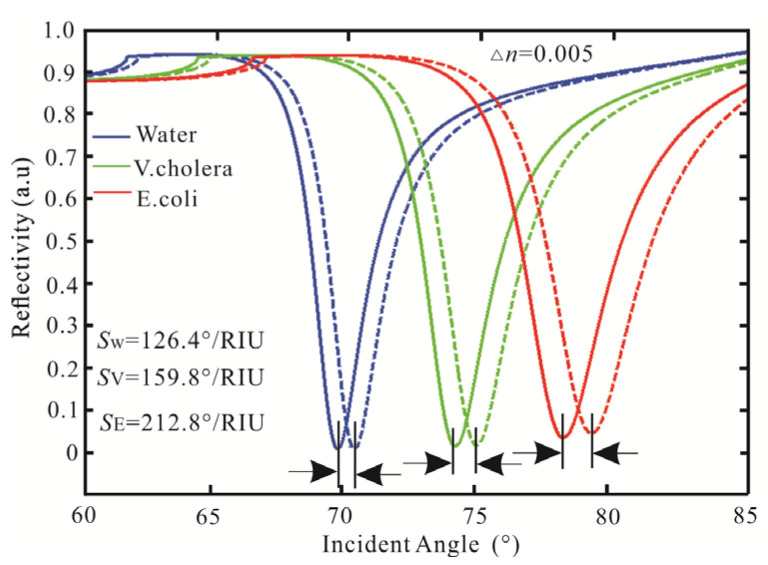
Variation of reflectivity concerning the incident angle for the SPR biosensor.

First, the variation to the incident angle for the SPR biosensor with an Ag-affinity-sensing medium structure is obtained in Figure 3, in which the sensing medium is △*n_s_* = 0.005. The sensitivity is adjusted by the thickness of the Ag thin film. When the thickness and affinity of Ag is 40 nm and 3 nm, the minimum reflectivity of pure water is 0.102 a.u, the Δ*θ* is 0.61°, and the sensitivity is 122°/RIU. With the same parameters as pure water, the minimum reflectivity of *V. cholera* is 0.093 a.u. At this time, the maximum Δ*θ* is 0.699°, and the sensitivity is 139.8°/RIU. When the sensing medium is *E. coli*, the least reflectivity is 0.069 a.u, the Δ*θ* is 0.952, and the sensitivity is 190.4°/RIU. Through the above analysis, with the increasing sensing medium refractive index, the reflectivity, Δ*θ*, and sensitivity are increasing.

**Figure 4:**
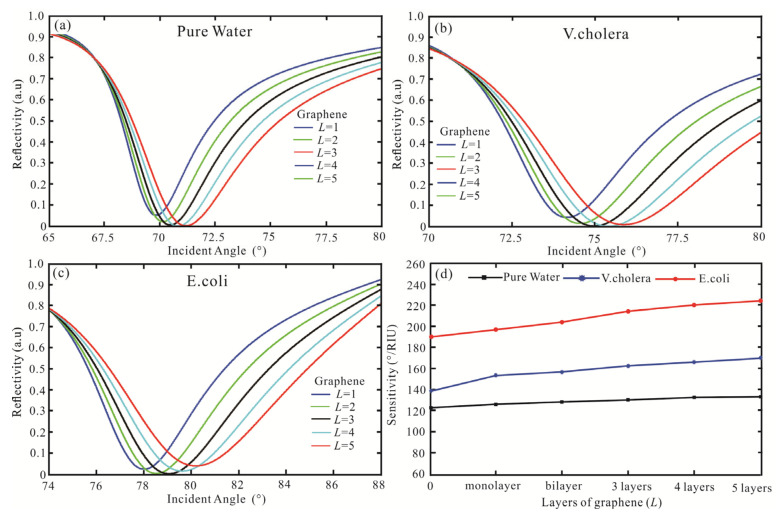
Variation of the reflectivity concerning the incident angle for the SPR biosensor with the structure of BK7/Ag/graphene/Affinity layer/sensing medium, (**a**) pure water, (**b**) *V. cholera*, (**c**) *E. coli*, (**d**) the sensing medium sensitivity for a different layer of graphene.

Subsequently, the SPR biosensor adds graphene to Ag film to obtain higher reflectivity and sensitivity. The reflectivity concerning the incident angle for the SPR biosensor is obtained as shown in Figure 4a–c. For Figure 4a of pure water, when the graphene monolayer is gained, the least reflectivity is 0.052 a.u (*θ* = 69.85°). When increasing the number of graphene layers, the reflectivity decreases, and the resonance angle increases. In the five graphene layers, the reflectivity is 2.4 × 10^−3^ a.u (*θ* = 71.2°). Then, the SPR biosensor with Ag-graphene-affinity layer-*V. cholera* is shown in Figure 4b. For the monolayer of graphene, the reflectivity is 0.043 a.u (*θ* = 74.16°). The optimal value is obtained in the third layer, the reflectivity is 1.8 × 10^−3^ a.u (*θ* = 74.98°). However, as the number of graphene layers continues to increase, the minimum reflectivity increases. The reflectivity obtained in the five graphene layers is 8.8 × 10^−3^ a.u, and the resonance angle is 75.89°. Finally, the SPR biosensor with Ag-graphene-Affinity layer-E coil is shown in Figure 4c. The minimum reflectivity is also three layers in graphene, and its reflectivity is 1.3 × 10^−3^ a.u (*θ* = 79.08°). When the maximum reflectivity occurs in the graphene five layer, the reflectivity is 0.039 a.u and the resonance angle is 80.22°. The sensing medium sensitivity for a different layer of graphene is shown in Figure 4d. For pure water, the sensitivity is 122°/RIU and FOM is 42.07 as no graphene. Then, the sensitivity and FOM are 124°/RIU and 30.06 as graphene monolayer, respectively. The sensitivity also increases graphene are 126.2°/RIU, 128.2°/RIU, and 130.6°/RIU, respectively. For five graphene layers, the sensitivity and FOM are 133°/RIU and 21.56, respectively. Therefore, the more layers of graphene are added to the traditional SPR biosensor, the sensitivity is increasing, while the FOM is decreasing. Then, for the *V. cholera*, when the graphene monolayer is added to the Ag-affinity structure, the sensitivity is 153.4°/RIU and FOM is 34.39. Nevertheless, the FOM is 29.77 (bilayer), 26.87 (three layers), 24.88 (four layers), and 23.72 (five layers), respectively. Finally, for the *E. coli*, in the graphene monolayer, the sensitivity and FOM are 197.8°/RIU and 37.18, respectively. The FOM is 33.18 (bilayer), 31 (three layers), and 29.85 (four layers), respectively. In the graphene five layers, the highest sensitivity is 225°/RIU, and the FOM is 29.45. Therefore, the Ag-affinity-graphene-sensing medium structure gradually increases, the sensitivity increases and the FOM decreases.

**Figure 5:**
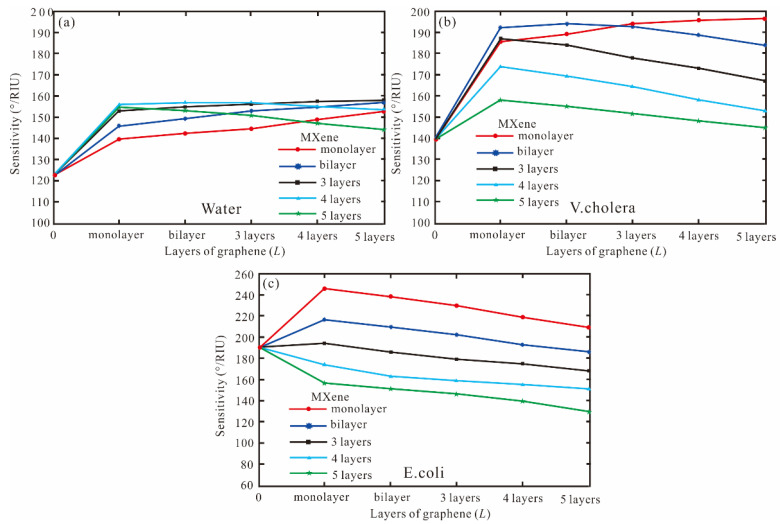
The sensitivity for a different layer of graphene and MXene, (**a**) pure water, (**b**) *V. cholera*, (**c**) *E. coli*.

Finally, the MXene is added to BK7/Ag/graphene/Affinity layer/sensing medium. For pure water, the sensitivity of the SPR biosensor for a different layer of graphene and MXene is shown in Figure 5a). For the MXene with monolayer to three layers, the sensitivity of graphene increases, which are 152°/RIU (monolayer), 157°/RIU (bilayer), and 158°/RIU (three layers), respectively. For the MXene four and five layers, the highest sensitivity is 156.4°/RIU and 154.6°/RIU, respectively. Therefore, the sensitivity with Ag-MXene-graphene-affinity-water structure is 158°/RIU for MXene three layers and graphene five layers. For the *V. cholera*, the sensitivity of Ag-MXene (1-5)-graphene (1-5)-affinity-*V. cholera* structure is shown in Figure 5b). When the MXene monolayer and graphene five layers are obtained, the sensitivity and FOM are 196.4°/RIU and 34.88, respectively. For the MXene bilayer, the sensitivity of 193.2°/RIU is obtained by the graphene bilayer. For the MXene from three to five layers, the sensitivity decreases. Therefore, the highest sensitivity of the SPR biosensor is 196.4 by Ag (62 nm)-MXene (monolayer)-graphene (five layers)-affinity (3 nm) structure. Finally, for the *E. coli*, when the MXene is increased from monolayer to five layers, the sensitivity decreases as Figure 5c). Therefore, graphene is a monolayer to obtain the best sensitivity. When the MXene and graphene are monolayers, the highest sensitivity, and FOM are 246.2°/RIU and 41.52, respectively. 

According to the above analysis, we can obtain the highest sensitivity and FOM of the thickness of Ag, MXene, graphene, and affinity to the SPR biosensor for detecting waterborne bacteria by FPS, as shown in Table 3 (the thickness of affinity is 3 nm).

## 4. The Principle of Improved Differential Evolution Algorithm

The DE algorithm has been proven to be a simple and effective evolutionary algorithm for real-world optimization problems. The theoretical research on differential evolution algorithms in China and abroad mainly focuses on the convergence analysis, computational complexity analysis, and spatial complexity analysis of the algorithm [45]. The performance of the DE algorithm mainly depends on three control parameters, namely hybrid probability *CR*, scaling factor *F*, and population size *NP*. It has insufficient local search ability, slow convergence speed, and low solution accuracy in dealing with high-dimensional problems. Although there are some achievements in the research on control parameter setting, the interaction parameter setting and optimization performance are complex and cannot be fully expressed [46]. The main reason is that no fixed parameters can be applied to all kinds of optimization problems, and even the setting of parameters in different optimization stages of a single problem has a great influence on the optimization performance of the algorithm. 

The IDE algorithm is proposed. The population size *NP* is analyzed, and the chaotic mapping method is applied to the DE algorithm, which mainly improves the quality of the initial population. The convergence speed and global search ability of the differential optimization algorithm are improved. The fitness elimination mechanism is adopted to recalculate the objective function values of the eliminated and replaced individuals. The eliminated and replaced populations are combined with the not eliminated populations to form the next iteration.

The pseudo-code of the IDE Algorithm 1 is as follows:
**Algorithm 1:** IDE algorithm Initialize:
(1) *G_m_*, *D*, *T*, *F*_0_, *CR*_max_, *CR*_min_, (2) population initialization particle *x*, mutation population *v*, selection population *u*, and target parameters *Ob*; **Cycle:** (3) **For** G = 1:1:G_m_ **do** (4) $Ob=S=func(x_{1,\ldots ,NP}^{i})$ (5) [Ob1,index]=sort(Ob) (6)     **For** m = 1:NP*T       $x_{index(m)}^{i}=\left(x_{index(NP-m+1)}^{i}+x_{index(NP-m)}^{i}\right)/T$       $Ob_{index(m)}^{i}=func(x_{index(m)}^{i})$ **End For** (7)     $F=F_{0}\cdot 2^{e^{1-\frac{G_{m}}{1+G_{m}-G}}}$ (8)   $CR=(CR_{max}-CR_{min}){\left(\frac{G}{G_{m}}\right)}^{2}+CR_{min}$ (9)  $v_{i}=x_{best}+F(x_{r1}-x_{r2}+x_{r3}-x_{r4})$        % DE/best/2/bin (10)       $u_{i,j}=\left\{\begin{array}{c} v_{i,j}\\ x_{i,j} \end{array}\begin{array}{c} if\;rand<CR\;or\;randi(1,D)=j\\ if\;rand>CR\;and\;randi(1,D)\neq j \end{array}\right.$ (11)       $x_{i,j}=\left\{\begin{array}{c} x_{\min}\\ \begin{array}{l} x_{\min}\\ x_{i,j} \end{array} \end{array}\begin{array}{c} if\;x_{i,j}<x_{\min}\\ \begin{array}{l} if\;x_{i,j}>x_{\max}\\ \mathrm{else} \end{array} \end{array}\right.$ (12)       **IF** (*fit*(*u_i_*) > *fit*(*x_i_*) **then**
*x_i_* = *u_i_*  **else**
*x_i_* = *x_i_*
(13)       **End If** (14)    **End For** (15) **End**

The IDE algorithm is used to optimize the sensitivity of the SPR biosensor. The thickness of Ag, MXene, graphene, and affinity and the angle of incident light are the overall coding factors.

## 5. The Optimization Multilayer SPR Biosensor Based on IDE

The objective function is relevant to the following geometric variables: (1) the thickness of Ag (*d*_1_), (2) the layer of MXene (*L*_2_), (3) the layer of graphene (*L*_3_), and (4) the thickness of affinity (*d*_4_). The following four design variables are, therefore:

(1) $$x={\left[d_{1},L_{2},L_{3},d_{4}\right]}^{T}={\left[x_{1},x_{2},x_{3},x_{4}\right]}^{T}$$

where the *x*_1_∈[1,100], *x*_2_∈[1,5], *x*_3_∈[1,5], and *x*_4_∈[1,10]. For *x*_4_ > 10 nm, the resonant dip vanishes. The IDE algorithm aims to optimize the sensitivity of SPR biosensors. The *NP* = 100, *F*_0_ = 0.1, *D* = 30, *CR*_min_ = 0.2, *CR*_max_ = 0.9, *T* = 0.3, and the mutation vector production mode as DE/best/2/bin are adopted. The objective function is the sensitivity of the SPR biosensor (*S*).

**Figure 6:**
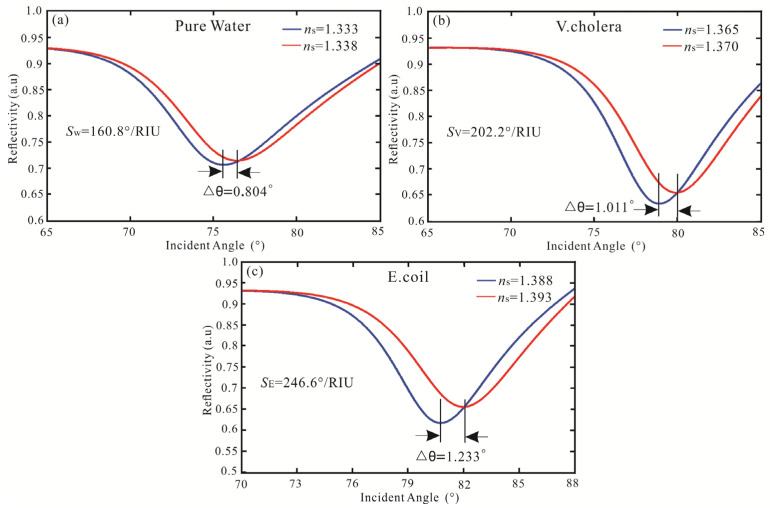
Variation of reflectivity concerning the incident angle for the SPR biosensor with the structure of (**a**) pure water, (**b**) *V. cholera*, and (**c**) *E. coli*.

The IDE algorithm is used to optimize sensitivity, FOM, and the number of iterations, as shown in Table 4. After three iterations, the highest sensitivity of the SPR biosensor with Ag-MXene-graphene-affinity-pure water hybrid structure is obtained. When the thickness of Ag and affinity are 62 nm and 10 nm, and the layers of MXene and graphene are bilayer and five layers, respectively, the maximum sensitivity is 160.8°/RIU and the FOM is 18.19. The sensitivity of pure water obtained by the FPS method is increased by 1.02 times and the FOM is increased by 1.14 times. Then, for the *V. cholera* by IDE algorithm, the optimal sensitivity and FOM are obtained after three iterations. When the SPR biosensor is Ag (61 nm), MXene (monolayer), graphene (three layers), and affinity (three), the highest sensitivity and FOM are 202.2°/RIU and 28.76, respectively. Compared with the FPS method, the sensitivity and FOM of the IDE algorithm are improved by 1.03 times, respectively. Finally, the IDE algorithm optimizes the SPR biosensor with an Ag-MXene-graphene-affinity-*E. coli* hybrid structure. After three iterations, the maximum sensitivity of 246.6°/RIU is obtained by MXene and graphene all monolayer. At the same time, the optimal FOM is 39.77. The sensitivity obtained by the IDE algorithm is similar to the FPS method. The IDE algorithm optimizes the highest sensitivity of the SPR biosensor, as shown in Figure 6.

**Figure 7:**
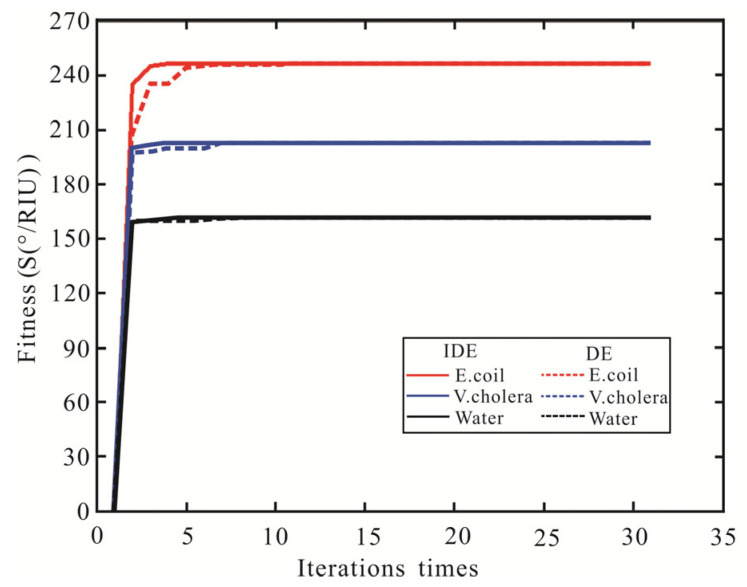
The variation curve of the sensitivity of SPR biosensor fitness value with the number of iterations.

Subsequently, the DE algorithm is proposed to optimize the sensitivity of the SPR biosensor. The parameters of the DE algorithm are set as follows: *NP* = 100, *D* = 30, *F* = 0.4, *CR* = 0.6, mutation vector production mode: DE/best/2/bin. The DE algorithm optimizes the sensitivity, FOM, and number of iterations for detecting waterborne bacteria are shown in Table 5. The sensitivity and FOM of the SPR biosensor obtained by the DE algorithm are the same as the IDE algorithm, but the iterations are eight times (Pure water), six times (*V. cholera*), and ten times (*E. coli*), respectively. The SPR biosensor Ag-MXene-graphene-affinity-sensing medium structure fitness of sensitivity with the number of iterations is shown in Figure 7. Compared to the DE algorithm, the IDE algorithm requires fewer iterations and has a stronger optimization ability. Therefore, The IDE algorithm has better convergence characteristics and optimization ability.

## 6. Comparative Analysis

In order to compare the results of previous studies, Table 6 summarizes the performance of metal-2D material-assisted SPR biosensors by the optimistic algorithm is made. In the designed SPR biosensor, the sensitivity has been significantly improved by the IDE algorithm.

For reference [27], the maximum sensitivity achieved by GA optimizing the refractive index of dielectric and the thicknesses of Ag and dielectric films is 228°/RIU, which is 3.17% higher than the one by optimizing the thickness of Ag and TiO_2_ films. For reference [31], when the graphene is monolayer, the highest sensitivity achieved by the PSO algorithm is 44°/RIU. For reference [33], after five iterations, the minimum fitness value is obtained by Ag-ITO-WS_2_-graphene structure, and the maximum sensitivity is 137.4°/RIU. 

## 7. Conclusions

In this paper, the BK7 prism-Ag-MXene-graphene-affinity-sensing medium of SPR biosensor is obtained and applied to the detection of waterborne bacteria. Using the FPS method, the maximum sensitivity of 246.2°/RIU is obtained by the MXene and graphene with monolayer, and sensing medium with *E. coli*. Subsequently, the IDE algorithm is improved to optimize the thickness of the multilayer SPR biosensor, to obtain the optimal sensitivity. After three iterations of the IDE algorithm, the maximum fitness value (sensitivity) of the SPR biosensor is 246.6°/RIU by Ag-MXene (monolayer)-graphene (monolayer)-affinity-*E. coli* hybrid structure. Compared to the DE algorithm, the IDE algorithm requires fewer iterations and has a stronger optimization ability. This paper provides an accurate, fast, and efficient platform for the performance optimization of multilayer SPR biosensors.

## Figures and Tables

**Table 1:** The monolayer and refractive index of 2D materials at the wavelength of 632.8 nm.

Type of 2D Materials	Monolayer (nm)	Refractive Index
MXene	0.993	2.38 + 1.33*i*
Graphene	0.34	3.0 + 1.1491*i*

**Table 2:** The refractive index of pure water, *V. cholera*, and *E. coli* at the wavelength of 632.8 nm.

Waterborne Bacteria	Refractive Index
Pure water	1.333
*V. cholera*	1.365
*E. coli*	1.388

**Table 3:** The different thicknesses of SPR biosensors for detecting waterborne bacteria with sensitivity and FOM.

Waterborne Bacteria	Ag (nm)	MXene (Layer)	Graphene (Layer)	Affinity (nm)	Sensitivity (°/RIU)	FOM
Pure water	57	3	5	3	158	15.96
*V. cholera*	62	1	5	3	196.4	34.88
*E. coli*	63	1	1	3	246.2	41.52

**Table 4:** The IDE algorithm optimizes the sensitivity, FOM, and iterations for detecting waterborne bacteria.

Waterborne Bacteria	Ag (nm)	MXene (L)	Graphene (L)	Affinity (nm)	Sensitivity (°/RIU)	FOM	Iterations (Times)
Pure water	62	2	5	10	160.8	18.19	4
*V. cholera*	61	1	3	10	202.2	28.76	3
*E. coli*	61	1	1	4	246.6	39.77	3

**Table 5:** The DE algorithm optimizes the sensitivity, FOM, and iterations for detecting waterborne bacteria.

Waterborne Bacteria	Ag (nm)	MXene (L)	Graphene (L)	Affinity (nm)	Sensitivity (°/RIU)	FOM	Iterations (Times)
Pure water	62	2	5	10	160.8	18.19	8
*V. cholera*	61	1	3	10	202.2	28.76	6
*E. coli*	61	1	1	4	246.6	39.77	10

**Table 6:** Comparison with SPR biosensor of sensitivity by the optimistic algorithm.

Multilayer Structure	MXene (Layers)	Graphene (Layers)	Sensitivity (°/RIU)	Optimistic Algorithm	References
Ag-TiO_2_-graphene	-	monolayer	228	GA	[27]
Au- graphene	-	monolayer	44	PSO	[31]
Ag-ITO-WS_2_-graphene	-	monolayer	137.4	PSO	[33]
Ag-MXene-graphene	monolayer	monolayer	246.6	IDE	This work

## Data Availability

Not applicable.
